# Viral load monitoring and antiretroviral treatment outcomes in a pediatric HIV cohort in Ghana

**DOI:** 10.1186/s12879-016-1402-9

**Published:** 2016-02-03

**Authors:** Omobolawa Kukoyi, Lorna Renner, Jonathan Powell, Oliver Barry, Meghan Prin, Jonas Kusah, Xiangyu Cong, Elijah Paintsil

**Affiliations:** 1 From Duke University School of Medicine, Durham, NC USA; 2 University of Ghana School of Medicine and Dentistry, Accra, Ghana; 3 Weill Cornell Medical College, New York, NY USA; 4 Boston Children’s Hospital, Harvard Medical School, Boston, MA USA; 5 Columbia University, New York, NY USA; 6 Yale Center for Analytical Sciences, Yale School of Medicine, New Haven, CT USA; 7 Departments of Pediatrics, Pharmacology & Public Health, Yale University School of Medicine, 464 Congress Avenue, New Haven, CT 06520 USA

**Keywords:** Pediatric HIV, Cumulative viral load, viral copy years, Morbidity, Resource-limited setting

## Abstract

**Background:**

HIV-infected children in sub-Saharan Africa may be at a high risk of staying on a failing first-line regimen and developing drug-resistance HIV variants due to lack of routine viral load monitoring. We investigated whether cumulative viral load, measured as viremia copy-years (VCY) could predict morbidity in a setting where viral load is not routinely monitored.

**Methods:**

This was a single-center prospective observational longitudinal study of HIV-infected children initiating antiretroviral therapy (ART) at the Pediatric HIV/AIDS Care program at Korle-Bu Teaching Hospital in Accra, Ghana. The main outcome was morbidity measured as frequency of hospitalizations, opportunistic infections, and outpatient sick visits. The main explanatory variable was viral load measured as VCY.

**Results:**

The study included 140 children who initiated ART between September 2009 and May 2013 and had at least 2 viral load measurements. There were 184 hospitalizations, with pneumonia being the most common cause (22.8 %). A total of 102 opportunistic infections was documented, with tuberculosis being the most common opportunistic infection (68 %). A total of 823 outpatient sick visits was documented, with upper respiratory infections (14.2 %) being the most common cause. Forty-four percent of our study participants had >4 log_10_ VCY. Children in this sub-cohort had a higher frequency of sick visits compared with those with <4 log_10_ VCY (*p* = 0.03). Only 6.5 % of children with >4 log_10_ VCY had been identified as treatment failure using WHO clinical and immunological treatment failure criteria.

**Conclusions:**

High level of cumulative viral load may translate to virological failure and subsequent increased all-cause morbidity. Our finding of potential utility of VCY in pediatrics warrants further investigations. VCY may be a good alternate to routine viral load measurement as its determination may be less frequent and could be personalized to save cost.

## Background

The advent of antiretroviral therapy (ART) has reduced HIV-associated morbidity and mortality in HIV-infected individuals. The benefits of ART have been substantial in sub-Saharan Africa, home to over 90 % of children living with HIV/AIDS [1]. Unfortunately, with the unprecedented scale-up of ART in resource-limited countries and the success of prevention of mother to child transmission (PMTCT) of HIV, pediatric HIV ART coverage still lags behind that of adults. At the end of December 2013, only 23 % of the 3.2 million children estimated to be living with HIV were receiving ART [2]. The World Health Organization (WHO) recently revised its recommendation on when to start ART in children; ART should be initiated among all children (<10 years of age) and adolescents (10 to 19 years) living with HIV, regardless of WHO clinical stage or CD4 cell count [3]. Based on this new recommendation, ART coverage in children living with HIV will increase sharply in the next couple of years.

Increase in ART coverage will exacerbate the existing challenges with laboratory monitoring of ART in resource-limited countries. In resource-rich countries, ART is monitored routinely with laboratory measures such as blood chemistry, HIV viral load, and CD4 count for early detection of medication side-effects and drug-resistant viruses [4, 5]. The WHO recommendation for ART monitoring has evolved over time from CD4 monitoring every six months and viral load testing only when the capacity exists [6] to viral load being recommended as the preferred monitoring approach to diagnose and confirm treatment failure [3]. However, if viral load is not routinely available, CD4 count and clinical monitoring should be used to diagnose treatment failure [3]. This compromise is due to the fact that routine laboratory monitoring is not feasible in most resource-limited countries due to cost, lack of technical expertise, and lack of infrastructure [7]. Several studies from sub-Saharan Africa have reiterated the need for universal access to viral load monitoring of ART as clinical and immunologic monitoring of treatment failure are not sensitive enough [8–12]. We recently reported that the rate of virological treatment failure after at least 24 weeks on first-line regimen was 16.7 % in our pediatric HIV cohort in Ghana [13]. The virological treatment failure rate in HIV-infected children receiving ART in sub-Saharan ranges from 13 % to 44 % [14–16].

In many resource-limited settings, clinicians rely on clinical and immunologic criteria to identify children failing first-line therapy. These criteria have low sensitivity and positive predictive value of detecting virological failure [11, 17]. Thus without viral load monitoring of ART, HIV-infected children in sub-Saharan Africa may be at increased risk of staying on a failing first-line regimen and developing drug-resistant HIV variants [18–20]. Viral load is therefore an essential complementary test to CD4 cell count [21] in monitoring treatment. The question, therefore, is not whether to do viral load testing in resource-limited countries but what is the most cost-effective way of monitoring viral load. Routine viral load monitoring (every 3–4 months) in resource-rich countries over 20 years was found to have limited additional health benefit considering the cost [22]. Taken together, there is a need to explore different cost-effective viral load measures such as less frequent, targeted, viral load measurements (based on clinical and immune response to ART) and cumulative viral load.

Given the grave consequences of virological treatment failure both at the individual and population level, we designed an exploratory prospective observational study of HIV-infected children receiving care at Korle-Bu Teaching Hospital in Accra, Ghana, to study biomarkers for monitoring ART in children [13, 23]. The main objectives of the current study were: (1) to evaluate the association between cross-sectional viral load and frequency of morbidity while on ART; and (2) to explore whether cumulative viral load, measured as viremia copy-years (VCY) [24], could predict morbidity in a setting where viral load is not routinely monitored.

## Methods

### Study population

This was a single-center prospective observational longitudinal study of HIV-infected children initiating ART at the Pediatric HIV/AIDS Care program at Korle-Bu Teaching Hospital in Accra, Ghana, from September 2009 to May 2013. Korle-Bu Teaching Hospital (KBTH) is the largest tertiary hospital in Ghana. The Pediatric HIV/AIDS Care program provides comprehensive HIV/AIDS care to over 1100 children. All study participants were on their first-line regimen. The first-line regimen available at the clinic is non-nucleoside analog (NNRTI)-based ART consisting of zidovudine (AZT), lamivudine (3TC), plus either nevirapine (NVP) or efavirenz (EFV).

The rationale, organization, and recruitment of the subjects for the cohort study have been previously described [13]. In brief, we enrolled children aged 0 to 13 years whose biological parents or guardians agreed to participate in a longitudinal study. Written consents from parents or guardians and assents from children were obtained before enrollment in the study. The participants were seen and examined at the pediatric HIV clinic every 4–6 months in accordance with local standard-of-care. At enrollment and subsequent follow-up visits, blood samples were collected for determination of CD4 cell count/percentage, HIV viral load, and complete blood count. The study was reviewed and approved by the Ethics and Protocol Review Committees of University of Ghana Medical School and Yale University School of Medicine.

### Study variables

#### Outcome variables

The main outcome measure was morbidity; measured as frequency of hospitalizations, opportunistic infections, and outpatient sick visits.

#### Explanatory variables

The main explanatory variable was viremia copy-years (VCY). VCY was defined as the number of copies of HIV RNA/ml/year [24]. Other variables included, gender, age, caregiver type, parental HIV status, history of AIDS defining illness, duration of ART, and WHO clinical staging of HIV infection.

### Other study measures

#### CD4 cell count and HIV viral load measurements

CD4 absolute cell count and percentage were quantified by a dual-platform flow cytometry technology using a FACSCount system (Becton-Dickinson, Franklin Lakes, NJ, USA) at the clinical laboratory at Korle-Bu Teaching Hospital (KBTH) according to manufacturer’s instructions. HIV viral load was measured using the COBAS® AmpliPrep and the COBAS® TaqMan® 48 Analyzer (Roche Diagnostic Systems, NJ, USA) for automated sample preparation and amplification and detection, respectively. The limit of HIV-1 RNA detection was 20 copies/mL. The laboratory participates in an external quality assurance testing program by the South African Public Health Reference Laboratory.

### Definitions

#### WHO clinical treatment failure

WHO defines clinical failure as a new or recurrent clinical event indicating advanced or severe immunodeficiency (WHO clinical stage 3 and 4 clinical condition with exception of TB) after 6 months of effective ART [25].

#### WHO immunological treatment failure

WHO defines immunological failure as a CD4 count of <200 cells/mm^3^ or CD4 < 10 % for children under 5; and a CD4 count of <100 cells/ mm^3^ for children above five years of age [25].

#### *WHO virological treatment failur*e

WHO defines virological treatment failure as plasma viral load above 1000 copies/ml based on two consecutive viral load measurements after 3 months of ART, with adherence support [25].

### Statistical analysis

Descriptive measures were used to summarize the data. Continuous variables were summarized using mean and standard deviation (SD); categorical variables were summarized using frequency and percent (%). Morbidity was measured using frequency of hospitalizations, opportunistic infections, and outpatient encounters. VCY was calculated as described above. A log_10_ transformation of each viral load measurement was performed and the area under the curve (AUC) was calculated. Participants were stratified into 3 categories: those with <2log_10_ VCY (low VCY), 2–4 log_10_ VCY (intermediate VCY) and >4log_10_ VCY (high VCY). Wilcoxon Rank Sum test and Fisher's exact tests were used to compare continuous and categorical variables, respectively, among the groups. Regression analyses using logistic regression models were used to estimate the odds of having low, intermediate or high VCY. Two-sided *p*-values are reported for all the statistical tests used in the analysis. Analyses were performed using SAS 9.3 (Cary, NC).

## Results

### Characteristics of study participants

We included 140 children who initiated ART between September 2009 and May 2013 with at least 2 viral load measurements. There were no loss to follow up during the study period. Table 1 summarizes the demographic characteristics of the study group. Of the 140 children, 56 (40 %) were female and 84 (60 %) were male. The primary caregiver for the children in this study was predominantly the biological mother (62 %) but varied considerably and included aunt (10 %), uncle (4 %), father (9 %), grandmother (13 %), and stepmother (2 %).

**Table 1:** Demographic characteristics of study participants

Characteristics	Frequency
	*N* = 140(%)
Age group (years)	
≤5	18 (12.85)
6–12	95 (67.86)
13–17	27 (19.29)
Gender	
Male	84 (60 %)
Female	56 (40 %)
HIV status of mother	
Positive	93 (66.43)
Negative	4 (2.86)
Unknown	43 (30.71)
HIV status of father	
Positive	34 (24.29)
Negative	17 (12.14)
Unknown	89 (63.57)
Caregiver	
Aunt	14(10.00)
Father	12 (8.57)
Grandmother	18 (12.85)
Mother	87 (62.14)
Stepmother	3 (2.14)
Uncle	6 (4.28)

### Morbidity among study participants

**Fig. 1:**
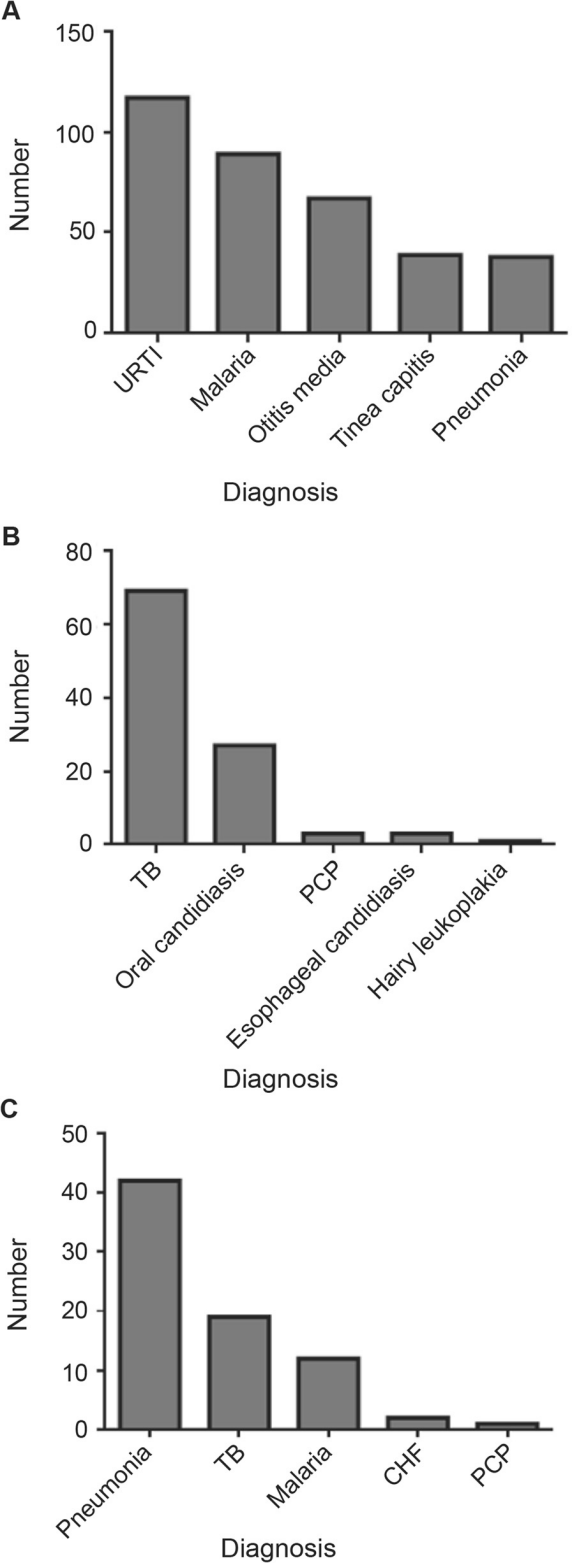
Top-five causes of morbidity among our pediatric cohort. **a**. Five most common diagnoses for outpatient sick visits. **b**. Five most common opportunistic infections. **c**. Five most common diagnoses for hospitalization

Morbidity was measured as frequency of hospital admissions, opportunistic infections, and outpatient sick visits. There were a total of 184 hospital admissions, with pneumonia – clinical and radiological diagnosis - being the most common cause (22.8 %). A total of 102 events of opportunistic infections was documented, with TB being the most common (68 %). A total of 823 outpatient sick visits was documented within the group, with upper respiratory infections (14.2 %). Figure 1 illustrates the five most common diagnoses for each of the morbidity measures.

### Association between viral load and morbidity

**Fig. 2:**
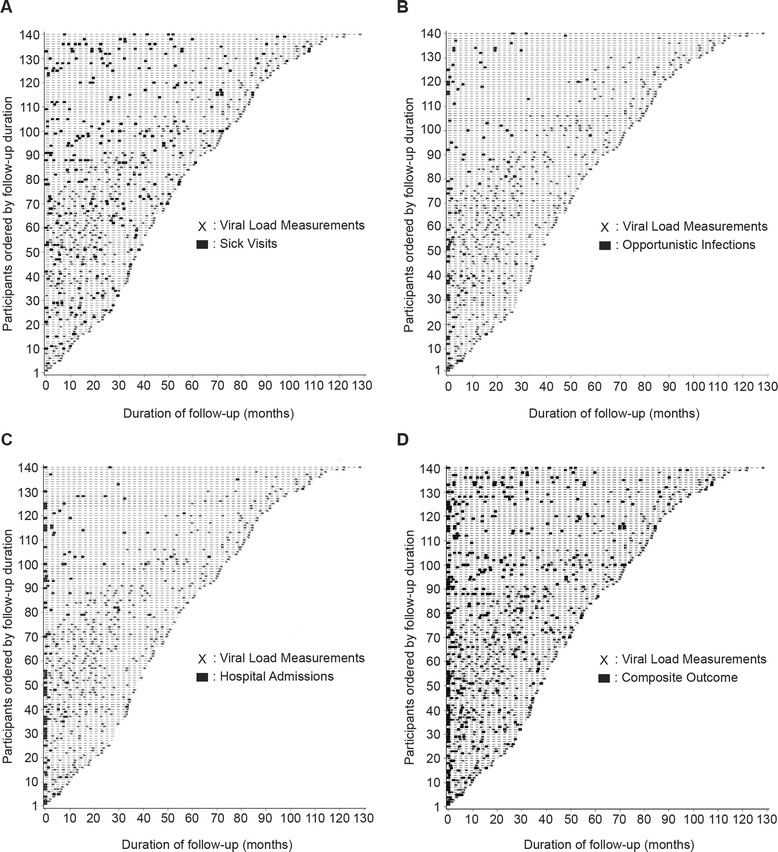
Time plots of viral load and morbidity among our pediatric cohort. Each follow up visit is represented with a dash; X is follow up visit where viral load was measured; dark rectangle represent outcome measure. **a**. Outpatient sick visits. **b**. Opportunistic infections. **c**. Hospitalization. **d**. Composite outcome; sum of hospitalization, opportunistic infections and outpatient sick visits

The mean follow up time was 4.3 years (SD ± 2.4). The median number of viral load measurements was 2 with a range of 2 to 8 measurements. We did not have uniform number of viral load measurements among study participants due to periodic shortages of reagents and technical difficulties with the viral load machine. We assessed whether there was any correlation between the number of viral load measurements and the outcome measures of interest (i.e., frequency of hospitalizations, opportunistic infections, and outpatient sick visits. There was no significant correlation between number of viral load measurements and any of the outcome measures. We then analyzed the temporal relationship between viral load and the outcome measures (Fig. 2). As illustrated in the time plots, the number of viral loads increased steadily with time for each participant.

### Viremia copy-year as predictor of morbidity

We investigated if any of the demographic characteristics of the study participants could predict cumulative viral load, measured as log_10_ VCY. Table 2 illustrates demographic characteristics of the study participants stratified into three groups based on log_10_ VCY; participants with <2 log_10_ VCY (*N* = 51), 2 to 4 log_10_ VCY (*N* = 27), and > 4 log_10_ VCY (*N* = 62). There was no statistically significant association between any of the demographic characteristics and VCY. Moreover, WHO clinical stage of a participant could not predict the level of VCY.

**Table 2:** Baseline demographic characteristics of study participants stratified by Log10 copy-years/mL

Characteristics	Less than 2 AUVLC(Log10 copy-years/mL) (*N* = 51)	Between 2 and 4 AUVLC(Log10 copy-years/mL) (*N* = 27)	Greater than 4 AUVLC(Log10 copy-years/mL) (*N* = 62)	*P* value^*^
Age	4.6 (2.9, 51)^a^	4.1 (3.0, 27)	5.4 (3.1, 62)	0.1327
HIV status of father				0.4012
Yes	14 (73.7)^b^	1 (33.3)	19 (65.5)	
No	5 (26.3)	2 (66.7)	10 (34.5)	
HIV status of mother				0.5237
Yes	36 (97.3)	18 (100.0)	39 (92.9)	
No	1 (2.7)	0 (0.0)	3 (7.1)	
Gender				0.2792
Female	16 (31.4)	13 (48.1)	27 (43.5)	
Male	35 (68.6)	14 (51.9)	35 (56.5)	
Care Giver				0.4816
Aunt	4 (7.8)	2 (7.4)	8 (12.9)	
Father	3 (5.9)	5 (18.5)	4 (6.5)	
Grandmother	4 (7.8)	3 (11.1)	11 (17.7)	
Mother	36 (70.6)	17 (63.0)	34 (54.8)	
Stepmother	1 (2.0)	0 (0.0)	2 (3.2)	
Uncle	3 (5.9)	0 (0.0)	3 (4.8)	
WHO clinical Stage				0.9904
Stage I	6 (21.4)	2 (13.3)	6 (14.6)	
Stage II	6 (21.4)	4 (26.7)	9 (22.0)	
Stage III	10 (35.7)	6 (40.0)	17 (41.5)	
Stage IV	6 (21.4)	3 (20.0)	9 (22.0)	

^*^The *p* value was generated using Wilcoxon Rank Sum test and Fisher’s Exact test for continuous and categorical variables, respectively

^a^Mean (STD, N) for continuous variables

^b^Number, N(%)for categorical variables

We investigated whether VCY was associated with outcome measures of interest and other HIV disease characteristics. Table 3 illustrates the bivariate analyses of disease characteristics of the study group stratified by VCY. There was no statistically significant difference in the number of years of follow-up among the 3 groups. We did not find any significant association between VCY and the number of opportunistic infections, or hospital admissions. However, the group with >4 log_10_ VCY had statistically significant outpatient sick visits compared with the other 2 groups (*p* = 0.03). Of note, only 6.5 % (4 of 58) in the group with >4 log_10_ VCY had been identified as treatment failure using WHO clinical and immunological definitions of treatment failure. There was no significant association between VCY and number of years of follow up.

**Table 3:** HIV disease characteristics of study participants stratified by Log10 copy-years/mL

Characteristics	Less than 2 AUVLC(Log10 copy-years/mL) (*N* = 51)	Between 2 and 4 AUVLC(Log10 copy-years/mL) (*N* = 27)	Greater than 4 AUVLC(Log10 copy-years/mL) (*N* = 62)	*P* value*
Overall Follow up time (in years)	4.6 (3.2, 51)^a^	4.6 (2.8, 27)	4.3 (1.7, 62)	0.96
AUVLC^b^ (copy-years/mL)	52839.09 (200451.75, 51)	334006.10 (963952.93, 27)	142793.89 (360264.57, 62)	
Number of opportunistic infections	0.7 (0.8, 51)	0.7 (0.6, 27)	0.8 (0.8, 62)	0.78
Number of outpatient sick visits	1.8 (2.1, 51)	2.3 (1.9, 27)	2.6 (2.3, 62)	0.08
Number of hospitalizations	0.9 (0.8, 51)	0.9 (1.1, 27)	0.8 (1.1, 62)	0.44
Number of composite events	3.6 (2.8, 140)	3.2 (2.5, 51)	3.7 (2.3, 27)	0.33
Clinical/Immunological Treatment Failure				0.49
Yes	2 (4.2)^c^	0 (0.0)	4 (6.5)	
No	46 (95.8)	26 (100.0)	58 (93.5)	
Opportunistic infections				0.90
Yes	27 (52.9)	16 (59.3)	35 (56.5)	
No	24 (47.1)	11 (40.7)	27 (43.5)	
Outpatient sick visits				0.03
Yes	33 (64.7)	22 (81.5)	53 (85.5)	
No	18 (35.3)	5 (18.5)	9 (14.5)	
Hospitalization				0.13
Yes	34 (66.7)	14 (51.9)	30 (48.4)	
No	17 (33.3)	13 (48.1)	32 (51.6)	
Composite Events^d^				0.39
Yes	47 (92.2)	27 (100.0)	57 (91.9)	
No	4 (7.8)	0 (0.0)	5 (8.1)	

*The *p* value was generated using Wilcoxon Rank Sum test and Fisher’s Exact test for continuous and categorical variables, respectively

^a^Mean (STD, N) for continuous variables

^b^AUVLC, area under viral load curve

^c^Number, N(%)for categorical variables

^d^Composite events, sum of hospitalization, opportunistic infections and outpatient sick visits

## Discussion

Forty-four percent of our study participants (62 of 140) had >4 log_10_ VCY. Interestingly, only 6.5 % of the participants with >4 log_10_ VCY were identified as meeting the WHO criteria for clinical and immunological treatment failures [25]. Moreover, participants with >4 log_10_ VCY had statistically significant increased outpatient encounters compared with participants with <4 log_10_ VCY. We found poor sensitivity of clinical and immunologic assessments in determining treatment failure, consistent with other pediatric studies from sub-Saharan Africa [8, 10, 11]. Taken together, our study validates the need for viral load monitoring as part of pediatric HIV care in resource-limited setting. Although implementation of viral load monitoring will continue to be constrained by budgetary and technical constraints in a resource-limited setting, there is an urgent need for innovative and cost-effective algorithms to include viral load monitoring for accurate and timely diagnosis of treatment failure.

We did not find statistically significant associations between cumulative viremia and frequency of opportunistic infections or hospitalizations during the study period. However, persistent viremia may have deleterious consequences with time. Interestingly, there are reports of an association between transient viremia and virological failure [26–28], depletion of CD4+ T-cells [29], and emergence of HIV drug resistant viruses [30, 31]. The hallmark of HIV infection is progressive depletion of CD4 T-cells leading to development of opportunistic infections (OIs), AIDS, and death [32]. HIV viral load has been found in several studies to be the main determinant of HIV disease progression [4, 33–35]. In a resource-limited setting, the two immediate deleterious effects of persistent viremia will be virological treatment failure and evolution of HIV drug resistant variants. Since the reported sensitivity of clinical/immunological monitoring in detecting treatment failure is low, 29 %-33 % [36], many of these children will continue to be on failing first-line regimens. Several studies have shown that delayed detection of virological failure to first-line therapy often leads to development of drug resistance, jeopardizing potential second-line treatments [11, 37, 38].

There is a paucity of data on the use of VCY, a measure of cumulative viremia, in monitoring ART in HIV-infected children. Two earlier pediatric studies found no association between cumulative viremia and virological failure or drug resistance [39, 40]. However, two recent studies reported findings consistent with our findings [41, 42]. Thorvaldsson et al. reported that for every 10-fold increase in viremia copy-years, there was about 8.5-fold increase in the risk of morbidity and mortality among a cohort of perinatally HIV-infected children in New Haven, CT, USA [41]. Rossouw et al. found that cumulative viremia in children on protease inhibitor (PI)-based ART was associated with development of PI resistant mutations [42]. In HIV-infected adults, there are several reports of significant association between VCY and morbidity and mortality [10–14]. Cole et al. reported that each log_10_ increase in VCY was associated with 1.70-fold increased hazard of AIDS or death among HIV seroconvertors, independent of duration of infection, age, race, CD4 cell count, viral load set-point, peak viral load, or most recent vial load [24]. The authors concluded that VCY is a better prognostic indicator of HIV disease progression than traditional single measures of viremia. Reports from other cohorts have reiterated that VCY is a better predictor of HIV all-cause mortality [43–45]. Taken together, viral load monitoring irrespective of the approach taken is critical for any HIV program. Therefore, the WHO in 2013 recommended viral load monitoring as the preferred approach in evaluating treatment failure [25].

The rationale for the new recommendation is to: (1) provide an early and more accurate detection of treatment failure and guide appropriate switch from first to second-line drugs to reduce the accumulation of drug-resistance mutations; and (2) help to differentiate between treatment failure and non-adherence [46]. However, there are several challenges involved in implementing these recommendations in resource-limited settings including costs of the test, costs of second-line drug regimens, accessibility to centers who offer the tests, and lack of laboratory infrastructure [47, 48]. For example, during our study period, we faced several occasions where there were no reagents to run viral load, breakdown of the equipment, technical problems, or power outages. These obstacles led to rescheduling patients for blood draws for viral load, and most patients subsequently failed to attend the rescheduled appointments due to lack of time or transportation. Despite the myriad of constraints in performing routine viral load monitoring in resource-limited settings, there is a need for countries to invest in innovative and cost-effective monitoring of HIV treatment. VCY may be a good alternate to routine viral load measurement or part of clinical/immunological criteria to improve sensitivity of detecting treatment failure. It also appears to be a good surrogate for persistent viremia, a determinant of HIV disease progression and non-AIDS-associated clinical events. To adopt VCY as part of treatment monitoring, there will be a need for studies to establish the “set-point” for VCY and how often to determine VCY. It may be possible to have multiple viral loads during the first year on therapy to determine one’s VCY “set-point” and thereafter the frequency could be personalized. Less frequent viral load determination might be cost-saving in the long-term.

Even though our study is one of the first studies to investigate the utility of VCY in monitoring ART in HIV-infected children, it has several limitations. First, this is a single tertiary center study and, therefore, one has to be cautious in generalization of our findings to all HIV care centers in resource-limited settings. Also, the small sample size limits the generalization of our findings. Moreover, viral load was not ascertained at regular interval so we could not adjust for transient viremia in the analysis. Due to periodic shortage of reagents or technical problems, viral load measurements were not available at each clinic visit or on scheduled dates. Since the viral loads were not uniformly missing at random among study participants we could not use any statistical test to account for data missing at random. Furthermore, data on adherence was not uniformly available in participants’ medical records so we could not model adherence in our analyses. Most of these limitations reflect the realities of carrying out observational cohort studies in a resource-limited setting. Our findings suggest potential utility of VCY in pediatrics, and this warrants further investigations in resource-limited settings.

## Conclusions

In conclusion, an unexpectedly high proportion of our study participants had increased cumulative viral load. This may translate to virological failure on their first-line regimen and subsequent increased all-cause morbidity. Our finding supports the current WHO recommendation of viral load monitoring as the preferred method for monitoring ART in resource-limited countries. HIV treatment programs in resource-limited countries should come up with innovative and cost-effective algorithms including viral load measurement for accurate and timely diagnosis of treatment failure. VCY may be a good alternate to routine viral load measurement as its determination may be less frequent and could be personalized and therefore a potential cost-saving approach.

## References

[1] UNAIDS. Global Report. UNAIDS report on the global AIDS epidemic 2013 [http://www.unaids.org/sites/default/files/en/media/unaids/contentassets/documents/epidemiology/2013/gr2013/UNAIDS_Global_Report_2013_en.pdf]. Accessed 1 Oct 2015.

[2] Optimizing treatment options and improving access to priority products for children living with HIV Brief [http://www.who.int/hiv/pub/toolkits/paediatric_art_optimisation-brief/en/]. Accessed 1 Oct 2015.

[3] World Health Organization WH. WHO Early Release Guidelines on when to start antiretroviral therapy and on pre-exposure prophylaxis for HIV September 2015. In. Geneva; 2015. [http://www.who.int/hiv/pub/guidelines/en/]. Accessed 1 Oct 2015. 26598776

[4] Mellors JW, Munoz A, Giorgi JV, Margolick JB, Tassoni CJ, Gupta P, Kingsley LA, Todd JA, Saah AJ, Detels R (1997). Plasma viral load and CD4+ lymphocytes as prognostic markers of HIV-1 infection. Ann Intern Med.

[5] Hammer SM, Saag MS, Schechter M, Montaner JS, Schooley RT, Jacobsen DM, Thompson MA, Carpenter CC, Fischl MA, Gazzard BG (2006). Treatment for adult HIV infection: 2006 recommendations of the International AIDS Society-USA panel. JAMA.

[6] World Health Organization Antiretroviral drugs for treating pregnant women and preventing HIV infection in infants: recommendations for public health approach. [http://whqlibdoc.who.int/publications/2010/9789241599818_eng.pdf.]. Accessed 20 August 2015. 26180894

[7] Taiwo BO, Murphy RL (2008). Clinical applications and availability of CD4+ T cell count testing in sub-Saharan Africa. Cytometry B Clin Cytom.

[8] Bolton-Moore C, Mubiana-Mbewe M, Cantrell RA, Chintu N, Stringer EM, Chi BH, Sinkala M, Kankasa C, Wilson CM, Wilfert CM (2007). Clinical outcomes and CD4 cell response in children receiving antiretroviral therapy at primary health care facilities in Zambia. JAMA.

[9] Hosseinipour MC, van Oosterhout JJ, Weigel R, Phiri S, Kamwendo D, Parkin N, Fiscus SA, Nelson JA, Eron JJ, Kumwenda J (2009). The public health approach to identify antiretroviral therapy failure: high-level nucleoside reverse transcriptase inhibitor resistance among Malawians failing first-line antiretroviral therapy. AIDS.

[10] Emmett SD, Cunningham CK, Mmbaga BT, Kinabo GD, Schimana W, Swai ME, Bartlett JA, Crump JA, Reddy EA (2010). Predicting virologic failure among HIV-1-infected children receiving antiretroviral therapy in Tanzania: a cross-sectional study. J Acquir Immune Defic Syndr.

[11] Ruel TD, Kamya MR, Li P, Pasutti W, Charlebois ED, Liegler T, Dorsey G, Rosenthal PJ, Havlir DV, Wong JK (2011). Early virologic failure and the development of antiretroviral drug resistance mutations in HIV-infected Ugandan children. J Acquir Immune Defic Syndr.

[12] Davies MA, Ford N, Rabie H, Fatti G, Stinson K, Giddy J, et al. Reducing CD4 Monitoring in Children on Antiretroviral Therapy with Virologic Suppression. Pediatr Infect Dis J. 2015. 10.1097/INF.0000000000000912 PMC5384719 26379169

[13] Barry O, Powell J, Renner L, Bonney EY, Prin M, Ampofo W, Kusah J, Goka B, Sagoe KW, Shabanova V (2013). Effectiveness of first-line antiretroviral therapy and correlates of longitudinal changes in CD4 and viral load among HIV-infected children in Ghana. BMC Infect Dis.

[14] Germanaud D, Derache A, Traore M, Madec Y, Toure S, Dicko F, Coulibaly H, Traore M, Sylla M, Calvez V (2010). Level of viral load and antiretroviral resistance after 6 months of non-nucleoside reverse transcriptase inhibitor first-line treatment in HIV-1-infected children in Mali. J Antimicrob Chemother.

[15] Adje-Toure C, Hanson DL, Talla-Nzussouo N, Borget MY, Kouadio LY, Tossou O, Fassinou P, Bissagnene E, Kadio A, Nolan ML (2008). Virologic and immunologic response to antiretroviral therapy and predictors of HIV type 1 drug resistance in children receiving treatment in Abidjan, Cote d'Ivoire. AIDS Res Hum Retrovir.

[16] Reddi A, Leeper SC, Grobler AC, Geddes R, France KH, Dorse GL, Vlok WJ, Mntambo M, Thomas M, Nixon K (2007). Preliminary outcomes of a paediatric highly active antiretroviral therapy cohort from KwaZulu-Natal, South Africa. BMC Pediatr.

[17] Jittamala P, Puthanakit T, Chaiinseeard S, Sirisanthana V (2009). Predictors of virologic failure and genotypic resistance mutation patterns in thai children receiving non-nucleoside reverse transcriptase inhibitor-based antiretroviral therapy. Pediatr Infect Dis J.

[18] Calmy A, Ford N, Hirschel B, Reynolds SJ, Lynen L, Goemaere E, Garcia de la Vega F, Perrin L, Rodriguez W (2007). HIV viral load monitoring in resource-limited regions: optional or necessary?. Clin Infect Dis.

[19] Sawe FK, McIntyre JA (2009). Monitoring HIV antiretroviral therapy in resource-limited settings: time to avoid costly outcomes. Clin Infect Dis.

[20] Mugyenyi P, Walker AS, Hakim J, Munderi P, Gibb DM, Kityo C, Reid A, Grosskurth H, Darbyshire JH, Ssali F (2010). Routine versus clinically driven laboratory monitoring of HIV antiretroviral therapy in Africa (DART): a randomised non-inferiority trial. Lancet.

[21] Harrigan R (1995). Measuring viral load in the clinical setting. J Acquir Immune Defic Syndr Hum Retrovirol.

[22] Phillips AN, Pillay D, Miners AH, Bennett DE, Gilks CF, Lundgren JD (2008). Outcomes from monitoring of patients on antiretroviral therapy in resource-limited settings with viral load, CD4 cell count, or clinical observation alone: a computer simulation model. Lancet.

[23] Renner L, Prin M, Li FY, Goka B, Northrup V, Paintsil E (2011). Time to and Predictors of CD4+ T-Lymphocytes Recovery in HIV-Infected Children Initiating Highly Active Antiretroviral Therapy in Ghana. AIDS Res Treat.

[24] Cole SR, Napravnik S, Mugavero MJ, Lau B, Eron JJ, Saag MS (2010). Copy-years viremia as a measure of cumulative human immunodeficiency virus viral burden. Am J Epidemiol.

[25] Consolidated Guidelines on the use of Antiretroviral Drugs for Treating and Preventing HIV Infection [http://www.who.int/hiv/pub/guidelines/arv2013/en/]. Accessed 20 Aug 2015.

[26] Grennan JT, Loutfy MR, Su D, Harrigan PR, Cooper C, Klein M, Machouf N, Montaner JS, Rourke S, Tsoukas C (2012). Magnitude of virologic blips is associated with a higher risk for virologic rebound in HIV-infected individuals: a recurrent events analysis. J Infect Dis.

[27] Zoufaly A, Kiepe JG, Hertling S, Hufner A, Degen O, Feldt T, Schmiedel S, Kurowski M, van Lunzen J (2014). Immune activation despite suppressive highly active antiretroviral therapy is associated with higher risk of viral blips in HIV-1-infected individuals. HIV Med.

[28] Lambert-Niclot S, Flandre P, Valantin MA, Peytavin G, Duvivier C, Haim-Boukobza S, Algarte-Genin M, Yazdanpanah Y, Girard PM, Katlama C (2011). Factors associated with virological failure in HIV-1-infected patients receiving darunavir/ritonavir monotherapy. J Infect Dis.

[29] Boufassa F, Saez-Cirion A, Lechenadec J, Zucman D, Avettand-Fenoel V, Venet A, Rouzioux C, Delfraissy JF, Lambotte O, Meyer L (2011). CD4 dynamics over a 15 year-period among HIV controllers enrolled in the ANRS French observatory. PLoS One.

[30] Cohen Stuart JW, Wensing AM, Kovacs C, Righart M, de Jong D, Kaye S, Schuurman R, Visser CJ, Boucher CA (2001). Transient relapses ("blips") of plasma HIV RNA levels during HAART are associated with drug resistance. J Acquir Immune Defic Syndr.

[31] Macias J, Palomares JC, Mira JA, Torres MJ, Garcia-Garcia JA, Rodriquez JM, Vergera S, Pineda JA (2005). Transient rebounds of HIV plasma viremia are associated with the emergence of drug resistance mutations in patients on highly active antiretroviral therapy. J Infect.

[32] Brady MT, Oleske JM, Williams PL, Elgie C, Mofenson LM, Dankner WM, Van Dyke RB, Pediatric ACTGCT (2010). Declines in mortality rates and changes in causes of death in HIV-1-infected children during the HAART era. J Acquir Immune Defic Syndr.

[33] O'Brien WA, Hartigan PM, Martin D, Esinhart J, Hill A, Benoit S, Rubin M, Simberkoff MS, Hamilton JD (1996). Changes in plasma HIV-1 RNA and CD4+ lymphocyte counts and the risk of progression to AIDS. Veterans Affairs Cooperative Study Group on AIDS. N Engl J Med.

[34] Mellors JW, Rinaldo CR, Gupta P, White RM, Todd JA, Kingsley LA (1996). Prognosis in HIV-1 infection predicted by the quantity of virus in plasma. Science.

[35] Saag MS, Holodniy M, Kuritzkes DR, O'Brien WA, Coombs R, Poscher ME, Jacobsen DM, Shaw GM, Richman DD, Volberding PA (1996). HIV viral load markers in clinical practice. Nat Med.

[36] Lynen L, Van Griensven J, Elliott J (2010). Monitoring for treatment failure in patients on first-line antiretroviral treatment in resource-constrained settings. Curr Opin HIV AIDS.

[37] Coetzer M, Westley B, Delong A, Tray C, Sophearin D, Nerrienet E, Schreier L, Kantor R (2013). Extensive drug resistance in HIV-infected Cambodian children who are undetected as failing first-line antiretroviral therapy by WHO 2010 guidelines. AIDS Res Hum Retrovir.

[38] Rawizza HE, Chaplin B, Meloni ST, Eisen G, Rao T, Sankale JL, Dieng-Sarr A, Agbaji O, Onwujekwe DI, Gashau W (2011). Immunologic criteria are poor predictors of virologic outcome: implications for HIV treatment monitoring in resource-limited settings. Clin Infect Dis.

[39] Lee KJ, Shingadia D, Pillay D, Walker AS, Riordan A, Menson E, Duong T, Tudor-Williams G, Gibb DM (2007). Collaborative HIVPSSC: Transient viral load increases in HIV-infected children in the U.K. and Ireland: what do they mean?. Antivir Ther.

[40] Hermankova M, Ray SC, Ruff C, Powell-Davis M, Ingersoll R, D'Aquila RT, Quinn TC, Siliciano JD, Siliciano RF, Persaud D (2001). HIV-1 drug resistance profiles in children and adults with viral load of <50 copies/ml receiving combination therapy. JAMA.

[41] Thorvaldsson OPE, Northrup V, Andiman W (2013). Cumulative viremai-copy years predicts morbidity and mortality in perinatally HIV-infected children. IDWeek.

[42] Rossouw TM, Feucht UD, Melikian G, van Dyk G, Thomas W, du Plessis NM, Avenant T (2015). Factors Associated with the Development of Drug Resistance Mutations in HIV-1 Infected Children Failing Protease Inhibitor-Based Antiretroviral Therapy in South Africa. PLoS One.

[43] Mugavero MJ, Napravnik S, Cole SR, Eron JJ, Lau B, Crane HM, Kitahata MM, Willig JH, Moore RD, Deeks SG (2011). Viremia copy-years predicts mortality among treatment-naive HIV-infected patients initiating antiretroviral therapy. Clin Infect Dis.

[44] Kowalkowski MA, Day RS, Du XL, Chan W, Chiao EY (2014). Cumulative HIV viremia and non-AIDS-defining malignancies among a sample of HIV-infected male veterans. J Acquir Immune Defic Syndr.

[45] Zoufaly A, Stellbrink HJ, Heiden MA, Kollan C, Hoffmann C, van Lunzen J, Hamouda O, ClinSurv Study G (2009). Cumulative HIV viremia during highly active antiretroviral therapy is a strong predictor of AIDS-related lymphoma. J Infect Dis.

[46] Orrell C, Harling G, Lawn SD, Kaplan R, McNally M, Bekker LG, Wood R (2007). Conservation of first-line antiretroviral treatment regimen where therapeutic options are limited. Antivir Ther.

[47] Belec L, Bonn JP (2011). Challenges in implementing HIV laboratory monitoring in resource-constrained settings: how to do more with less. Futur Microbiol.

[48] Roberts T, Bygrave H, Fajardo E, Ford N (2012). Challenges and opportunities for the implementation of virological testing in resource-limited settings. J Int AIDS Soc.

